# Diffusiophoretic Transport of Charged Colloids in Ionic Surfactant Gradients Entirely below versus Entirely above the Critical Micelle Concentration

**DOI:** 10.1021/acs.langmuir.4c00431

**Published:** 2024-05-01

**Authors:** Angela Yang, Brian E. McKenzie, Benjamin Pavlat, Eric S. Johnson, Aditya S. Khair, Stephen Garoff, Robert D. Tilton

**Affiliations:** † Department of Chemical Engineering, Carnegie Mellon University, Pittsburgh, Pennsylvania 15213, United States; ‡ The Procter & Gamble Company, Cincinnati, Ohio 45241, United States; § Department of Physics, Carnegie Mellon University, Pittsburgh, Pennsylvania 15213, United States; ∥ Department of Biomedical Engineering, Carnegie Mellon University, Pittsburgh, Pennsylvania 15213, United States

## Abstract

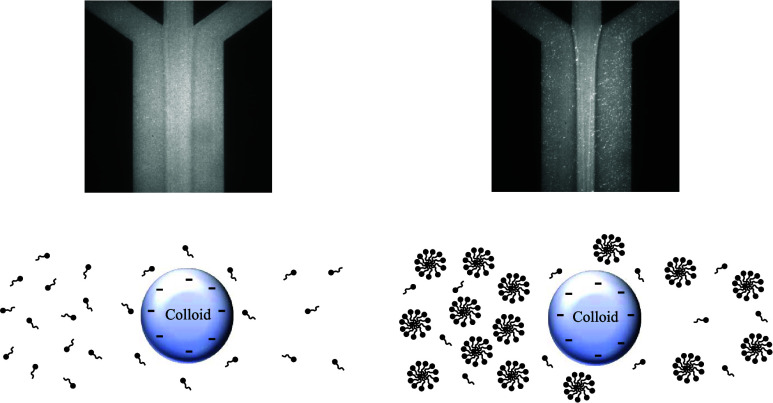

When placed in an ionic surfactant gradient, charged colloids will undergo diffusiophoresis at a velocity, *u*_DP_ = *M*_DP_∇ ln *S*, where *M*_DP_ is the diffusiophoretic mobility and *S* is the surfactant concentration. The diffusiophoretic mobility depends in part on the charges and diffusivities of the surfactants and their counterions. Since micellization decreases surfactant diffusivity and alters charge distributions in a surfactant solution, *M*_DP_ of charged colloids in ionic surfactant gradients may differ significantly when surfactant concentrations are above or below the critical micelle concentration (CMC). The role of micelles in driving diffusiophoresis is unclear, and a previously published model that accounts for micellization suggests the possibility of a change in the sign of *M*_DP_ above the CMC [WarrenP. B.; . Soft Matter2019, 15, 278–288]30534797 10.1039/c8sm01472h. In the current study, microfluidic channels were used to measure the transport of negatively charged polystyrene colloids in sodium dodecyl sulfate (SDS) surfactant gradients established at SDS concentrations that are either fully above or fully below the CMC. Interpretation of diffusiophoresis was aided by measurements of the colloid electrophoretic mobility as a function of SDS concentration. A numerical transport model incorporating the prior diffusiophoretic mobility model for ionic surfactant gradients was implemented to elucidate signatures of positive and negative diffusiophoretic mobilities and compare with experiments. The theoretically predicted sign of the diffusiophoretic mobility below the CMC was determined to be particularly sensitive to uncertainty in colloid and surfactant properties, while above the CMC, the mobility was consistently predicted to be positive in the SDS concentration range considered in the experiments conducted here. In contrast, experiments only showed signatures of a negative diffusiophoretic mobility for these negatively charged colloids with no change of sign. Colloid diffusiophoretic transport measured in micellar solutions was more extensive than that below the CMC with the same ∇ ln *S*.

## Introduction

From pharmaceuticals to consumer products, surfactants are routinely used in complex fluid formulations to control their stability, surface wetting, and delivery, among other critical performance metrics.^1−3^ Surfactant concentration gradients can arise naturally or by design in the application or manufacture of these formulations.^4^ In the presence of surfactant gradients, diffusiophoresis, the deterministic transport of colloids in solute gradients, may be the dominant colloidal transport mechanism and may be engineered to control the transport direction and rate in applications of such formulations.

The diffusiophoretic mobility, *M*_DP,_ establishes the proportionality between the diffusiophoretic colloid velocity and the driving force, which is the gradient of the logarithm of the solute concentration. For ionic solutes, including ionic surfactants, the mobility depends on the ζ-potential of the colloid, as well as on the ionic valences (*z*_1_ and *z*_2_) and the difference in the diffusivities of the anions and the cations (*D*_1_ and *D*_2_) parameterized by β, which for electrolytes is specified as .^5,6^ Due to the significant size mismatch between ionic surfactants and their counterions (often alkali metal cations or halide anions), the difference between the anion and cation diffusivities parameterized by β is often large.

Furthermore, above the critical micelle concentration (CMC), by assembling multiple surfactant ions and a fraction of their counterions, micellization alters the diffusivities and charge distributions in a surfactant solution, thereby altering diffusiophoresis in surfactant gradients. Additionally, depending on the surface chemistry of the colloids, surfactants may adsorb to the particles and alter their ζ-potential in a concentration-dependent manner. This in turn alters diffusiophoretic mobility so that its value may change across the surfactant gradient.

Experimental studies on surfactant-driven diffusiophoresis below the CMC show that general trends in diffusiophoresis in nonmicellar surfactant gradients are predictable by theoretical concepts established for diffusiophoresis in simple electrolyte gradients,^7−10^ yet there are phenomena in surfactant solutions that are not addressed in these models. The capacity for oppositely charged surfactant adsorption to reverse the net surface charge of a colloid has been considered previously, but the systematic variation of the colloid ζ-potential across a surfactant gradient has not been addressed in the quantitative interpretation of diffusiophoresis.^7^ Both Akdeniz and co-workers and Lee and co-workers have used principles of colloidal electrostatics to address the variation of diffusiophoretic mobility caused by changes in ζ-potential as a function of ionic strength in monovalent electrolyte gradients,^11,12^ but the effect of surfactant adsorption on ζ-potential leading to a variable diffusiophoretic mobility remains untested.

The explicit effects of ionic surfactant micellization on diffusiophoresis remain uncertain. Systems influenced by micellization have been investigated, as have systems where ionic surfactants form complexes with nonionic polymers.^7,13,14^ Experimentally, diffusiophoresis measurements in microfluidic devices with sodium dodecyl sulfate (SDS) gradients established by semipermeable membranes showed weaker diffusiophoretic transport above the CMC than below it.^7^ The authors proposed that this was due to minimal surfactant monomer concentration gradients, which they argued were the main solute gradient driving force, excluding the role of micelles from the driving force for transport.^7^ The authors noted that scatter in the measurements made it difficult to quantitatively interpret the mobility in solutions above the CMC. This motivated the subsequent development of a model to predict the effect of micellization on the diffusiophoretic transport of charged colloids in ionic surfactant gradients.^13^

That diffusiophoresis model incorporated the micellar surfactant diffusion model of Leaist in a detailed account of the electrophoretic and chemiphoretic components of the diffusiophoretic mobility to predict *M*_DP._(13,15) Using SDS and negatively charged colloids as an illustrative model system, the model predicted a negative diffusiophoretic mobility below the CMC, consistent with experimental observations, and an initial decrease in the magnitude of *M*_DP_ at the CMC, followed by a reversal in sign and a significant increase in the magnitude of *M*_DP_ at larger surfactant concentrations.^13^ The concentration at which the mobility reversed sign depended on the colloid ζ-potential, but this colloid ζ-potential was assumed to be constant throughout the surfactant concentration gradient. This prediction of a mobility reversal at increasing surfactant concentrations is a motivating factor underlying the current research.

The question this study aims to answer is do the direction and magnitude of diffusiophoretic colloid transport driven by surfactant gradients change in the presence of micelles, and if so, how? To do so, this study provides measurements of diffusiophoretic transport of negatively charged polystyrene colloids in gradients of the anionic surfactant SDS in a microfluidic device. Measurements are conducted in surfactant concentration gradients that are established either fully above or fully below the CMC, where the gradients of the logarithm of the surfactant concentration are the same (meaning they have the same 10-fold difference between the highest and lowest solute concentrations) in both cases. Experiments are complemented by numerical calculations of colloidal transport in the microfluidic device that establish the signatures of a negative or a positive diffusiophoretic mobility, incorporating the mobility model of Warren and co-workers,^13^ including calculations that explicitly account for the SDS concentration-dependent ζ-potential. The experimental results indicate that *M*_DP_ is negative below the CMC and remains negative without a sign reversal at concentrations that are well above the CMC and the concentrations at which a reversal is predicted. Additionally, the results indicate that diffusiophoretic transport is significant in both micellar and nonmicellar SDS solutions. This suggests that micelles and their counterions do, in fact, contribute significantly to diffusiophoresis of charged colloids.

## Background

The diffusiophoretic velocity, *u*_DP_, is
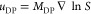
1where *S* is the solute concentration.^5^ The diffusiophoretic mobility can be divided into two parts *M*_DP_ = *M*_EP_ + *M*_CP_, where *M*_EP_ is the electrophoretic contribution based on the electrophoretic migration of the particle in response to the mismatch in diffusion coefficients between the charged solute species, and *M*_CP_ is the chemiphoretic contribution, which accounts for the effect of osmotic pressure differences in the double layer due to concentration gradients in the bulk. For a 1:1 electrolyte, Anderson, Prieve, and co-workers predicted the diffusiophoretic mobility contributions as
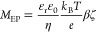
2a
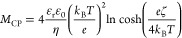
2bwhere ε_r_ε_0_ is the permittivity of the solvent, η is the viscosity of the solvent, *k*_B_*T*/*e* is the thermal voltage, ζ is the ζ-potential of the colloid, and the diffusivity mismatch parameter , where *D*_1_ and *D*_2_ are the diffusion coefficients of the anion and cation, respectively.^5^ Due to the constant ratio of cations and anions in a symmetric electrolyte, this expression for *M*_DP_ is independent of solute concentration. Additionally, a symmetric electrolyte diffuses with a constant ambipolar diffusion coefficient .^15^ However, in systems with more than two charged species, the expressions above are no longer valid. In fact, the changes in relative species concentrations in a system with micellization lead to a concentration dependence, in contrast to the expressions above.^13^ The existing theory developed by Warren and co-workers^13^ for the diffusiophoresis of a particle in a gradient of ionic surfactants undergoing micellization will now be summarized to establish key principles underpinning the current research.

The first step to modeling diffusiophoresis in a micellar solution is to determine the degree of micellization as a function of the total SDS concentration. Warren and co-workers describe a method for this by assuming fast relaxation time scales for micelle kinetics (≲0.1 s), leading to local equilibrium between monomers, counterions, and micelles, the size of which is assumed to be monodisperse and concentration-independent.^13^ Leaist originally developed a model for this called the quasi-chemical association model,^15^ which was employed by Warren and co-workers.^13^ Here, a micelle consists of *n* surfactant monomers and *q* counterions (*n* > *q*) so that it carries a negative charge of magnitude *n* – *q*. The concentrations (number densities) of monomers, counterions, and micelles, called *S*_1_, *S*_2_, and *S*_m_, respectively, are governed by mass conservation through *S* = *S*_1_ + *nS*_m_ = *S*_2_ + *qS*_m_ and by an equilibrium relation based on the law of mass action through *S*_m_ = *KS*_1_^*n*^*S*_2_^*q*^, where *K* is an equilibrium constant for micellization related to the critical micelle concentration, *S*_cmc_, as *K* = *n*^–1^*S*_cmc_^–*n*–*q*^. The fraction of SDS molecules incorporated into micelles, α, defined as
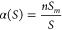
3must be found to obtain the equilibrium concentrations of monomers, counterions, and micelles at a given total surfactant concentration. This micellization fraction α(*S*) can be approximated (yielding the species concentrations *S*_1_, *S*_2_, and *S*_m_) as a stepwise function of the total surfactant concentration: for *S* ≤ *S*_CMC_, α = 0 due to lack of micelles, and for *S* > *S*_CMC_, α satisfies the transcendental equation
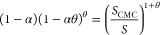
4as derived asymptotically in Appendix A of Warren and co-workers,^13^ where θ is the fraction of counterion binding to the micelle. The micellization fraction α(*S*) can then be determined numerically by solving eq 4 for α at a number of solute concentrations and interpolating.

Using the Nernst–Planck equations and enforcing no charge current, Warren and co-workers^13^ (following Leaist^15^) derive the electric field generated by the diffusivity mismatch between each solute species as
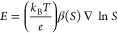
5where β(*S*) is now a concentration-dependent dimensionless diffusion-mismatch parameter. For the micellizing surfactant solution, β(*S*) is
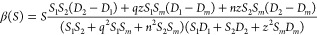
6where *D*_1_, *D*_2_, and *D_m_* are the self-diffusion coefficients of, respectively, surfactant monomers, counterions, and micelles. For SDS, β is always positive because sodium counterions have larger diffusivity than both surfactant monomers and micelles, and thus the sign of β does not depend on the relative concentrations of these two negatively charged species. Below the CMC, β reduces to the well-known constant expression for a 1:1 binary electrolyte: β_submicellar_ = (*D*_2_ – *D*_1_)/(*D*_1_ + *D*_2_). Satisfying the conservation equation ∂*S*/∂*t* = ∇·(*D*_S_(*S*)∇*S*), where *D*_S_ is the overall collective diffusion coefficient gives
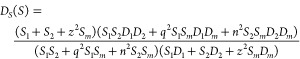
7Below the CMC, *D*_S_ reduces to the well-known constant ambipolar diffusivity *D*_S_ = 2*D*_1_*D*_2_/(*D*_1_ + *D*_2_), which is independent of *S*.^13^

The phenomena discussed in the previous paragraphs do not yet address the presence of a colloidal particle; now the diffusiophoretic migration of a colloidal particle in a gradient of micellizing surfactant will be discussed. In Warren and co-workers,^13^ the electrophoretic contribution uses eq 2a for *M*_EP_ replacing β with β(*S*) given by eq 6, accounting for micellization. Using eq 5 to substitute the electric field *E* in place of ∇ ln *S*, the expression for the particle velocity driven by *M*_EP_ is mathematically equivalent to the Smoluchowski expression for electrophoretic migration,^16^ as a consequence of the mutual assumptions of a thin double layer, a weak field, and negligible polarization. For negatively charged particles in SDS solutions, as occur in the current work, the sign of *M*_EP_ is always negative.

Above the CMC, Warren and co-workers^13^ assumed that the binary electrolyte Guoy–Chapman expression (based only on the monomer and counterion) for the electric potential field in the double layer around the particle was unaffected by micelles due to an anticipated depletion of highly negatively charged micelles within the double layer near the negatively charged particle. This yields the chemiphoretic contribution to the diffusiophoretic mobility as
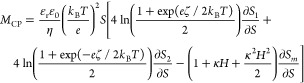
8where κ*H* = ln(2.852 *z* tanh|*e*ζ/4*k*_B_*T*|). This expression relies on the assumption that the micelle is negatively charged like the colloid particle surface, and it reduces to the concentration-independent eq 2b below the CMC (*S*_m_ = 0 and *S*_1_ = *S*_2_ = *S*). *M*_CP_ is always positive, driving particles up the solute gradient toward regions of higher solute concentration. In the valid region of moderately negative ζ-potentials, *M*_CP_ is also concave up with respect to the ζ-potential (as compared to the expression for *M*_EP_, which varies linearly with respect to the ζ-potential). Due to this feature, the relative magnitude of the chemiphoretic contribution compared to that of the electrophoretic contribution increases for larger magnitudes of the ζ-potential. This is the reason underlying the prediction that the positive chemiphoretic mobility eventually dominates the negative electrophoretic mobility leading to a sign reversal in *M*_DP_ above the CMC for negatively charged colloids at larger SDS concentrations.

Whereas the model of Warren and co-workers^13^ was derived based on a constant ζ-potential, the ζ-potential is known to change with solute concentration^14^ due to electrostatic effects in simple electrolyte solutions as well as the adsorption that is possible in ionic surfactant solutions. To account for the measured variation of the colloid ζ-potential with surfactant concentration in the gradient, a concentration-dependent expression based on fitting the measured values for the ζ-potential will be used, in addition to the original model with a constant ζ-potential for the sake of comparison, to predict diffusiophoretic transport in this work.

## Experimental Section

### Materials

Microfluidic channels were prepared as previously described by Yang and co-workers by casting poly(dimethylsiloxane) (PDMS) (Sylgard 184, Dow) on SU-8 epoxy (Kayaku Advanced Materials) molds on silicon wafers.^14,17^ Sodium dodecyl sulfate (SDS) (BioXtra grade, >99% purity) and sodium chloride (NaCl) were obtained from Sigma-Aldrich. One micrometer nominal diameter yellow/green carboxylate-modified fluorescent microspheres (FluoSpheres, Thermo Fisher Scientific, determined by dynamic light scattering as 1.1 ± 0.2 μm in intensity-weighted hydrodynamic diameter with *D*_p_, the colloid diffusivity, thus being (3.9 ± 0.3) × 10^–13^ m^2^/s) were suspended at a concentration of 0.005 vol % in aqueous solutions of either SDS or NaCl. The ζ-potential of the colloids in surfactant-free 0.1 mM NaCl solution was −65 ± 5 mV. Water was purified with a Milli-Q IQ 7003/05/10/15 Water Purification System to 18.2 MΩ·cm resistivity, and fresh SDS solutions were made the day before each experiment. All experiments were conducted at room temperature, 20 ± 1 °C.

### Methods

**Figure 1:**
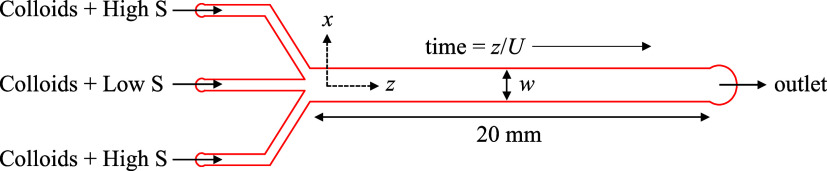
Three-inlet PDMS microfluidic device. The width, *w*, and depth of the channel were 1.5 mm and 85 μm, respectively. The inlet channel widths were each 0.5 mm. *U* was the average velocity in the channel, and *x* and *z* were the transverse and axial directions of the channel, respectively.

Diffusiophoresis experiments were conducted in three-inlet PDMS microfluidic channels illustrated schematically in Figure 1. The device was operated in a manner similar to that described previously, with the exception that here colloids were pumped at equal concentrations in each of the three inlet channels at the same concentration, whereas colloids were only introduced to the central inlet channel in the prior work.^14^ (This new arrangement was better for discerning signatures of negative or positive values of *M*_DP_ that might arise in different surfactant concentration ranges within the imposed gradient.) The colloid concentration was 0.005 vol % in all three inlet channels. The outer inlet channels contained equal, high-solute concentrations, while the central inlet channel contained a low-solute concentration to establish the solute gradient. The solute concentration gradient was thus symmetrical about the overall channel midline, *x* = 0.

Laminar flow was driven along the axial, *z*-direction by three matched syringe pumps (PHD 2000, Harvard Apparatus), serving each inlet channel with 1 cm diameter syringes (BD Luer Lock), at a flow rate of 1.0 μL/min for the experiments with SDS gradients and 1.0 or 0.5 μL/min for the experiments with NaCl gradients. Since diffusiophoresis in NaCl gradients is well understood, the latter experiments were conducted to aid in experiment validation. The average velocity in the channel, *U*, was
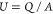
9where *Q* was the volumetric flow rate and *A* is the channel cross-sectional area. For *Q* = 1.0 or 0.5 μL/min, *U* was 1.3 × 10^–4^ or 6.5 × 10^–5^ m/s, respectively. Colloid transport was observed in the transverse *x*-direction at varying downstream positions *z*, corresponding to varying transport times, *t* = *z*/*U*.^18^ For this experimental configuration, it is important to note that while the channel is operated at steady state, solute concentrations vary with position, and the solute gradient across the channel relaxes as a function of axial distance downstream. This allows for the observation of the time-dependent evolution of the diffusiophoretic colloidal transport by recording the colloids in the channel at different axial distances down the microfluidic channel. Due to the parabolic nature of the flow field, it is noted that there is a 44% variance in axial velocity across the channel depth, so this time *z*/*U* represents the average time for colloid transport, while colloids at different depths experience different velocities, and thus different residence times.

A Nikon Eclipse Ti-U microscope with a moving stage was used to image the fluorescent colloidal particles in the microfluidic cell. The whole length of the cell was captured by a set of positions that spanned the length of the cell, with 100 images taken 1 second apart at each position and averaged. By imaging at different axial positions along the 20 mm main channel in this way, transport was observed over times up to 140 s. Comparing images at the same axial position (same colloidal transport time *t*) at early and late actual times after the onset of the experiment ensured that all recorded concentration profiles in the channel were at steady state. ImageJ (National Institutes of Health) was used to analyze line scans of average fluorescence intensities in the transverse direction of the channel. Local intensities were normalized by the total area under the intensity line scan curves. Verification of the working colloid concentration range of the detection system, which established direct proportionality between fluorescence intensity and colloid concentration, as well as channel flow assumptions, was reported previously.^14^

ζ-Potential measurements of negatively charged polystyrene particles in varying SDS concentrations were performed using a Zetasizer Nano ZS (Malvern). A manufacturer-provided standard was measured before and after each set of measurements to validate the measurement cell. The Smoluchowski equation was used to convert measured electrophoretic mobilities to ζ-potentials. Dynamic light scattering measurements confirming the intensity-weighted hydrodynamic diameter of the colloids were performed using a Zetasizer Nano ZS (Malvern).

## Numerical Simulations of Diffusiophoretic Transport

Diffusiophoretic colloid transport in the channel for all cases was computed by solving the solute and colloid transport equations numerically. Using a similar procedure to Yang and co-workers,^14^ the diffusion equation for the transient one-dimensional solute transport where *t* = *z*/*U* is
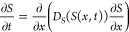
10Here, the solute diffusivity, *D*_S_, is the collective diffusion coefficient and varies as a function of the local surfactant concentration due to micellization. The diffusion–migration equation describing colloid transport by both diffusion and diffusiophoretic migration at a velocity, *u*_DP_, is
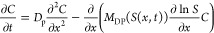
11where *C* is the colloid concentration and *D*_p_ is the colloid particle diffusivity.^19,20^*M*_DP_ varies as a function of the local solute concentration.

The solute and colloid profiles (satisfying eqs 10 and 11, respectively) are solved on half the channel width (0 ≤ *x* ≤ *h*), where *h* = *w*/2 = 0.75 mm and then symmetrically mirrored onto the other half 0 ≤ *x* ≤ – *h* due to the symmetry of the channel about the centerline *x* = 0. Therefore, no-flux boundary conditions at the channel center (*x* = 0) and the channel edge (*x* = *h*) are applied
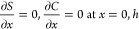
12

The colloid concentration initial condition is represented by a uniform concentration *C*_0_ (=0.005 vol %) in all three inlets. The solute concentration initial condition is expressed as a smoothed step change in concentration as used previously^14,21,22^
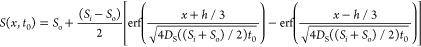
13where *S*_0_ and *S*_i_ are the initial concentrations of the solute in the outer and inner channels, respectively. To avoid the initial discontinuity in solute concentration at *t*_0_ = 0 s, the problem is solved starting at *t*_0_ = 0.01 s. The time before this is a miniscule fraction of the times (of order 100 s) over which the concentration profiles evolve in the experiments. Mathematica’s NDSolve function is used to solve eqs 10–13 and obtain the solute and colloid concentration profiles from *t* = *t*_0_ to *t* = 140 s on 0 ≤ *x* ≤ *h* and then, as stated above, the results are symmetrically mirrored onto 0 ≤ *x* ≤ – *h*.

## Results and Discussion

### Sensitivity of Sign of Mobility to Uncertainty in Inputs above and below the CMC

The main contributing factor to a predicted reversal in sign for the diffusiophoretic mobility of a negatively charged colloid in SDS solutions is the competition between the positive chemiphoretic and negative electrophoretic contributions to the diffusiophoretic mobility. Uncertainty in the input parameters to these contributions that alter the relative magnitude of each may thus have significant effects on the prediction of the sign of the diffusiophoretic mobility. Therefore, this section begins by examining the effect of uncertainty in the model input parameters, based on the variation in reported values that appear in the literature, on the predicted sign of the diffusiophoretic mobility.

As will be discussed below, in all cases, there is some sufficiently high SDS concentration, above which the diffusiophoretic mobility is predicted to be positive. A threshold concentration *S*_X_ will be defined as the highest SDS concentration for which *M*_DP_ = 0, with *M*_DP_ becoming positive for *S* > *S*_X_. The set of input parameters, which yield the highest values of *S*_X_, will be shown to be *n* = 60, θ = 0.84, *D*_DS–_ = 3.9 × 10^–10^ m^2^/s, and *D_m_* = 1.0 × 10^–10^ m^2^/s, and these values will be used by default for further calculations unless otherwise noted. This practice was adopted to give a conservative treatment, affording the greatest opportunity for the model to predict a negative *M*_DP_.

The default input for the ζ-potential will be a concentration-dependent expression fitted to the measured colloid ζ-potentials, as discussed below. In some cases, constant ζ-potential values representing extremes just outside the measured range will also be tested to check the robustness of the conclusions regarding the comparison of model and experimental results. A complete list of tested combinations and resulting values of *S*_X_ is given in Table S1 of the Supporting Information. After considering all possible values for *S*_X_, it will be concluded that *M*_DP_ is predicted to be exclusively positive for SDS concentrations in the experimentally relevant concentration range of 30–300 mM SDS.

#### Uncertainty in Permittivity and Viscosity

Though it may be expected that permittivity and viscosity of the surrounding fluid will change with SDS concentration, these terms serve as prefactors to the diffusiophoretic mobility, modifying its magnitude without modifying the competition between the electrophoretic and chemiphoretic contributions. Therefore, the changes in permittivity and viscosity will have no effect on the sign of the mobility or the concentration at which the mobility changes sign.

#### Uncertainty in Critical Micelle Concentration, Aggregation Number, and Fraction of Counterion Binding

In the quasi-chemical association model of Leaist,^15^ three values are used to characterize micellization: the critical micelle concentration, *S*_CMC_, the aggregation number of the micelle, *n*, and the fraction of counterion binding, θ. Varying the CMC, recognizing the approximate 5% range of variability in reported CMC values for SDS,^23−25^ will simply rescale the dependence of the fraction of micellization on the solute concentration, as can be seen in eq 4. Thus, the effect of varying *S*_CMC_ is a slight proportional shift in the trends to be discussed below (including the value of *S*_X_) with respect to the solute concentration, with higher values of *S*_CMC_ leading to proportionally higher values of *S*_X_. A value of *S*_CMC_ = 8.2 mM^23,24^ will be used in all cases.

**Figure 2:**
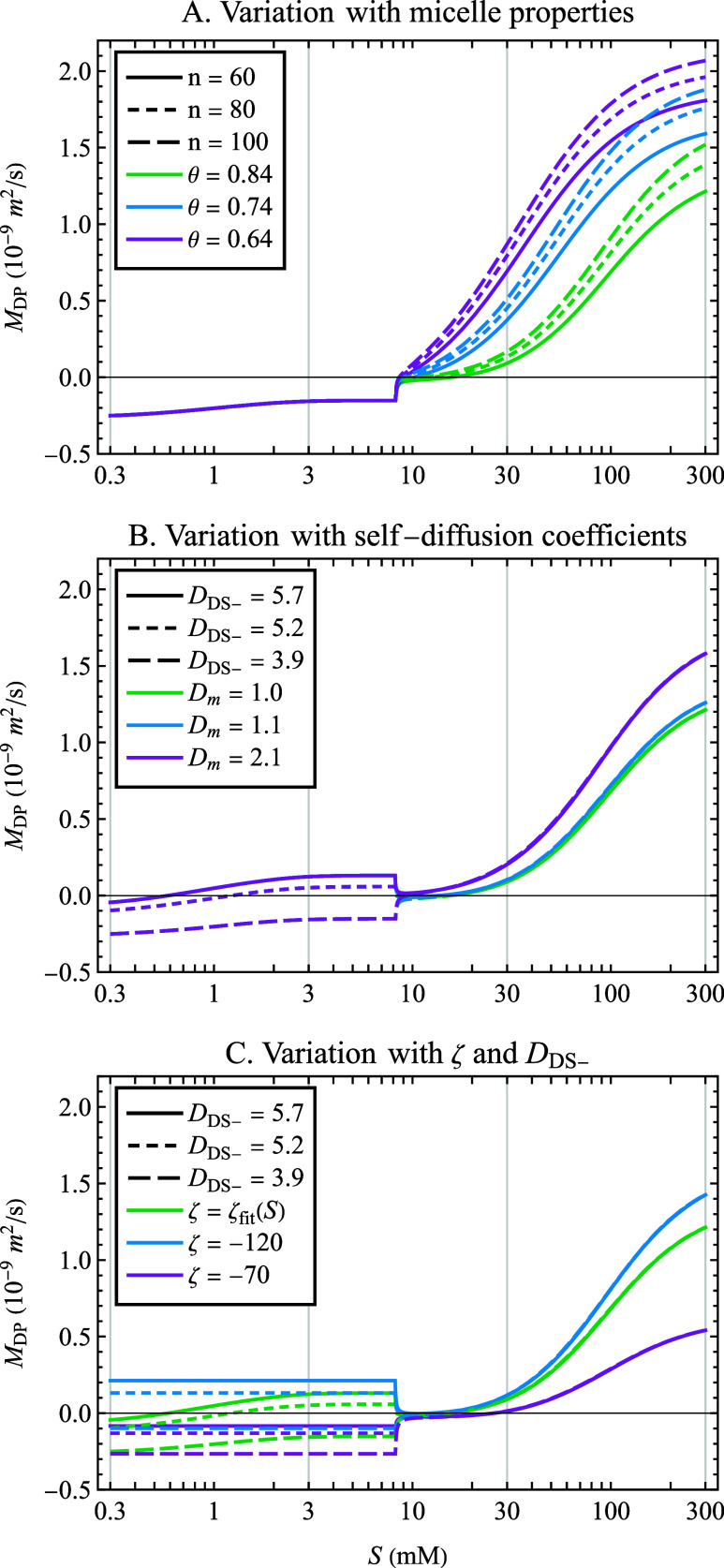
Predicted diffusiophoretic mobility dependence on SDS concentration, *S*, with variation in (A) micelle aggregation number, *n*, and fraction of counterion binding, θ; (B) self-diffusion coefficients of dodecyl sulfate ion, *D*_DS_^–^, and micelles, *D_m_*, (10^–10^ m^2^/s); and (C) ζ-potential (mV) coupled with variation in dodecyl sulfate ion, *D*_DS_^–^, self-diffusion coefficient (10^–10^ m^2^/s).

To determine the sensitivity to the aggregation number, *n*, the model was evaluated at values of *n* = 60,^13,26^ 80,^25^ and what should be considered an extreme value of 100 based on the variability of values in the literature. The fraction of counterion binding θ was varied between 0.64, 0.74, and 0.84.^13,26^ The nine combinations of these pairs of *n* and θ were used to calculate *M*_DP_ as a function of *S*, and the results are plotted in Figure 2A. There is no effect of micelle properties below the CMC for obvious reasons. The effect of varying the aggregation number *n* was weak, with higher values favoring a more positive *M*_DP_. The effect of varying θ was stronger, with lower values of θ favoring a more positive *M*_DP_.

Overall, variation in *n* and θ was not strong enough to significantly change the SDS concentration at which the predicted mobility changed sign. For all of these justifiable combinations of *n* and θ, the diffusiophoretic mobility changed sign from the negative value exhibited below the CMC to positive values at a threshold concentration somewhere above the CMC. Among these cases using the fitted concentration-dependent ζ-potential, the lowest threshold concentration was *S*_X_ = 8.6 mM for *n* = 100 and θ = 0.64, and the highest threshold was *S*_X_ = 15.7 mM for *n* = 60 and θ = 0.84 (the values used in calculations by Warren and co-workers^13^). The complete range of *S*_X_ values under all input conditions is included in Table S1 of the Supporting Information. For the sake of analyzing the conditions most likely to predict a negative diffusiophoretic mobility above the CMC, all following calculations will use *n* = 60 and θ = 0.84, which are seen to predict the least positive diffusiophoretic mobility values in the experimental concentration range.

#### Uncertainty in Solute Self-Diffusion Coefficients (*D*_1_, *D*_2_, and *D_m_*)

The following values are reported by Warren and co-workers^13^ for the surfactant monomer self-diffusion coefficient *D*_DS_^–^ (used as *D*_1_ for SDS): *D*_DS_^–^ = 5.7 × 10^–10^,^13,27^ 5.2 × 10^–10^,^28^^28^ and 3.9 × 10^–10^ m^2^/s. ^8^^8^ Self-diffusion coefficients for SDS micelles have been reported as *D_m_* = 2.1 × 10^–10^,^14^ 1.0 × 10^–10^, and 1.1 × 10^–10^ m^2^/s.^13^*D*_Na_^+^ = 1.3 × 10^–9^ m^2^/s is used for *D*_2_, where Na^+^ denotes the sodium counterion, in all calculations. While electrolyte self-diffusion coefficients do exhibit concentration dependence at high concentrations due to interactions between like ions,^29,30^ this effect on *D*_Na_^+^ is negligible compared to the larger variability in reported values of *D*_DS_^–^ and *D_m_*.

The effect of all nine combinations of the three values of *D*_DS_^–^ and the three values of *D_m_* on the diffusiophoretic mobility is shown in Figure 2B, with *S*_X_ values reported in Table S1 of the Supporting Information. Below the CMC, *D_m_* has no effect for obvious reasons, but uncertainty in *D*_DS_^–^ has a large effect on the diffusivity mismatch parameter β, leading to changes in the predicted sign of the diffusiophoretic mobility across the values tested. Above the CMC, the diffusiophoretic mobility is far more sensitive to *D_m_* than to *D*_DS–_. This reflects the fact that at high SDS concentrations, the micelle replaces the monomer as the most prominent anionic species. Nevertheless, in the tested region above the CMC, all combinations of diffusion coefficients still cause the electrophoretic contribution to be dominated by the chemiphoretic contribution, and the diffusiophoretic mobility is predicted to be positive for all SDS concentrations exceeding 15.7 mM, the highest value of *S*_X_ among these conditions, found when *D*_DS–_ = 3.9 × 10^–10^ m^2^/s and *D_m_* = 1.0 × 10^–10^ m^2^/s. These self-diffusion coefficients maximize the diffusivity contrast parameter β and thus also maximize the strength of the negative electrophoretic contribution (with no effect on the positive chemiphoretic contribution *M*_CP_). Therefore, this set of values is used in subsequent calculations unless stated otherwise.

#### Uncertainty in the ζ-Potential

The final source of uncertainty to be considered is the ζ-potential, which has a strong effect on both the electrophoretic and chemiphoretic contributions to the diffusiophoretic mobility. Up to this point, all justifiable combinations of CMC, aggregation number, degree of counterion binding, and self-diffusion coefficients predict positive diffusiophoretic mobilities above the CMC in the relevant experimental range. To determine whether uncertainty in ζ-potential could lead to predictions of negative diffusiophoretic mobilities in this range, the ζ-potential was varied and the values for previously discussed properties in this section that gave the weakest propensity to generate positive diffusiophoretic mobilities, in other words, to be most permissive of a negative diffusiophoretic mobility, were used. Thus, *n* = 60, θ = 0.84, *D*_DS–_ = 3.9 × 10^–10^ m^2^/s, and *D_m_* = 1.0 × 10^–10^ m^2^/s were used.

**Figure 3:**
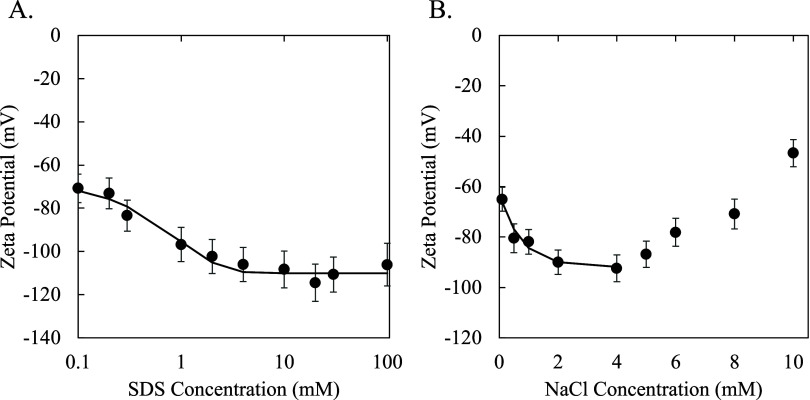
Polystyrene colloid ζ-potentials and their fits using eq 14 for (A) varying SDS concentration (log scale) or (B) varying NaCl concentration (linear scale).

Since a control experiment was conducted with the 1:1 electrolyte NaCl (known to yield a positive diffusiophoretic mobility for a negatively charged colloid), Figure 3 shows the measured ζ-potentials of negatively charged polystyrene latex particles versus SDS (Figure 3A) and NaCl (Figure 3B) concentration, along with fits of the measured values that take the form of an exponential function as
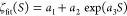
14where *a*_1_, *a*_2_, and *a*_3_ are the fitting parameters with values of *a*_1_ = −109 mV, *a*_2_ = 42.4 mV, and *a*_3_ = −1.21 mM^–1^ for SDS and *a*_1_ = −91.9 mV, *a*_2_ = 29.9 mV, and *a*_3_ = −1.36 mM^–1^ for NaCl. The NaCl data is only fitted from 0.1 to 4 mM NaCl because the concentration in the NaCl-driven diffusiophoresis experiments ranges only from 0.3 to 3 mM. Figure 3A shows an increasingly negative ζ-potential value with increasing SDS concentration. This was due to SDS adsorption onto the surface of the colloids, which increased the negative surface charge density of the colloids up until adsorption reached its maximum extent at the CMC, and the ζ-potential plateaued as a result. Figure 3B shows that the ζ-potential initially became more negative with NaCl concentration increasing from 0.1 to 4 mM. A maximum in the magnitude of the ζ-potential is reached at 4 mM NaCl followed by a decrease in magnitude with further increases in NaCl concentration. This trend is consistent with previous ζ-potential measurements of negatively charged polystyrene colloids in NaCl solutions.^31^

The model predictions reported by Warren and co-workers^13^ held the ζ-potential constant for all SDS concentrations. Here, the dependence of the ζ-potential on SDS concentration is incorporated into the expression for mobility. Figure 2C compares *M*_DP_ predicted using the concentration-dependent ζ-potential ζ_fit_(*S*) to predictions made by assuming a constant ζ-potential value. The two constant values chosen for comparison, ζ = −70 and −120 mV, represent extremes as they lie just outside the measured range for suspensions in SDS solutions. Three different values were considered for *D*_DS_^–^.

Below the CMC, calculations using constant ζ-potential values yielded constant diffusiophoretic mobilities, in accordance with eqs 2a and 2b. However, the concentration-dependent ζ_fit_(*S*) caused the diffusiophoretic mobility to vary even below the CMC, including a change in sign from negative to positive in this range for some cases. The effect of the concentration dependence of the ζ-potential was most prominent below the CMC. Above the CMC, the measured ζ-potential became constant, with the fit ζ_fit_(*S*) asymptotically approaching a value of −109 mV. In all cases, more negative values of the ζ-potential lead to more positive values of *M*_DP_. This is because the expression for *M*_CP_ has a nonlinear (concave up) dependence on ζ in the relevant range, while the dependence on ζ of *M*_EP_ is linear; thus, at large magnitudes of ζ, *M*_CP_ (which is strictly positive in sign) becomes larger than *M*_EP_.

The calculation with the constant value ζ = −70 mV yielded the highest threshold concentration *S*_X_ = 25.9 mM due to the relative weakness of *M*_CP_ relative to *M*_EP_ as compared to more strongly negative ζ-potentials. Because all justifiable combinations of input parameters predicted a positive value of the diffusiophoretic mobility for SDS concentrations exceeding *S*_X_ = 25.9 mM, the surfactant concentration gradient selected for experiments conducted above the CMC was set to 300–30–300 mM SDS (where concentrations indicate the high SDS concentrations in the outer inlets and the low SDS concentration in the central inlet of the microfluidic channel). Although ζ = −70 mV yields the highest value of *S*_X_, the more realistic concentration-dependent ζ_fit_(*S*) will be used for the ζ-potential in all other calculations unless otherwise noted to capture the known concentration dependence of the ζ-potential.

Another source of uncertainty in the ζ-potential is discussed by Nery-Azevedo and co-workers^7^ where the retarding effect of concentration polarization on the measured particle electrophoretic speed for moderate to high values of the ζ-potential (ζ ≳ 4*k*_B_*T*/*e*) is unaccounted for in the ζ-potentials reported in Figure 3. Correcting for this effect, ζ-potentials would be higher in magnitude than the computed values from the Smoluchowski expression.^7^ However, since the Zetasizer Nano ZS (Malvern) instrument measures the electrophoretic mobility directly, the electrophoretic contribution to diffusiophoresis *M*_EP_ is, in effect, measured directly. Therefore, if the ζ-potential given by the Smoluchowski expression mispredicts the true ζ-potential, the discrepancy is eliminated when this ζ-potential is used in *M*_EP_, due to the mathematical equivalence of the particle velocities driven by *M*_EP_ and the Smoluchowski expression, as discussed above. Thus, there is high confidence that the theoretically predicted value of the electrophoretic contribution *M*_EP_ given by the measured ζ-potential reflects the true effect in the measured diffusiophoretic transport, and as is seen in Figure 2C, a larger magnitude negative ζ-potential favors positive diffusiophoretic mobility.

In summary, below the CMC, the diffusiophoretic mobility can change sign due to relevant variations in both ζ and *D*_DS_-, as these parameters have the greatest impact on the predicted sign of the diffusiophoretic mobility. Above the CMC, the highest threshold concentration above which the diffusiophoretic mobility was positive was *S*_X_ = 25.9 mM. Therefore, all variations in input parameters (micelle properties, self-diffusion coefficients, and ζ-potentials) lead to a positive diffusiophoretic mobility that increases with SDS concentration in the concentration range of 30–300 mM that was selected for experiments with gradients established entirely above the CMC. The set of parameters chosen to maximize the possibility of a negative diffusiophoretic mobility in this range lead to higher threshold concentrations *S*_X_ than are shown in Figure 10 of Warren and co-workers,^13^ but similar trends were otherwise observed above the CMC.

The computational results above established that the existing theory consistently predicts positive diffusiophoretic mobilities in the high-concentration range that was selected for experiments, for all reasonable variation in input parameters. Next, the transport eqs 10–13 were solved numerically with different input parameters to predict colloid concentration profiles in the experimental microfluidic geometry, with particular focus on varying the ζ-potential input, which was the input of greatest impact on the sign of the diffusiophoretic mobility. This will establish signatures of a changing sign of *M*_DP_. These would be recognizable patterns in colloid concentration profiles that would develop in the experiments.

**Figure 4:**
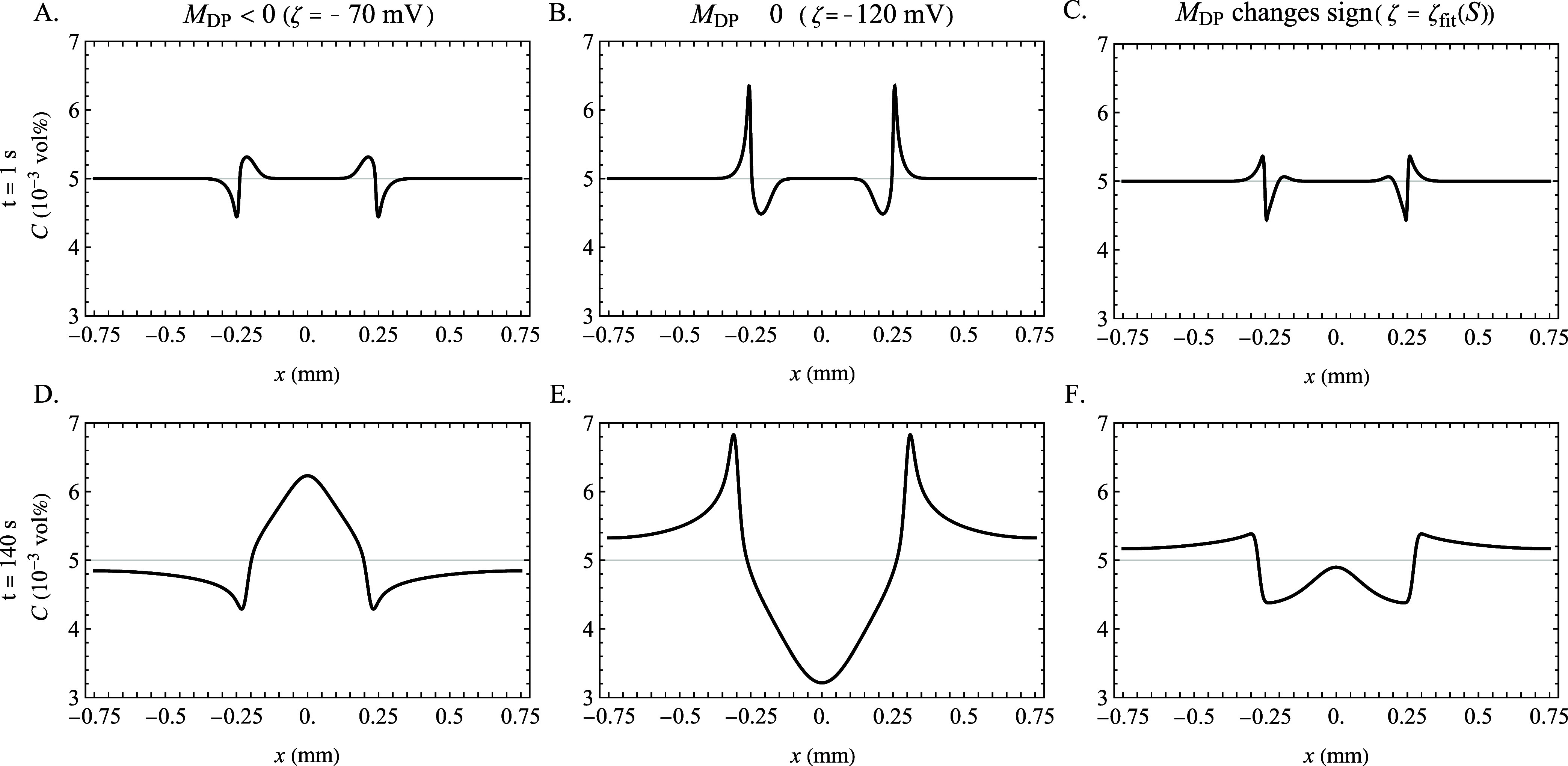
Numerically computed colloid concentration profiles at *t* = 1 s (A–C) and *t* = 140 s (D–F) for solute concentration gradient 3–0.3–3 mM SDS, entirely below the CMC. The value *D*_DS–_ = 5.7 × 10^–10^ m^2^/s and three ζ-potentials were chosen to illustrate the effect of the sign of the mobility on the colloid profile: (A, D) ζ = −70 mV yields a constant negative mobility (B, E) ζ = −120 mV yields a constant positive mobility. (C, F) ζ = ζ_fit_(*S*) yields a mobility which switches from negative to positive at *S* > 0.59 mM SDS. Other input parameters used were *S*_cmc_ = 8.2 mM, *n* = 60, θ = 0.84, *D*_Na+_ = 1.3 × 10^–9^ m^2^/s, and *D_m_* = 1.0 × 10^–10^ m^2^/s.

First, to establish these signatures, the equations were solved below the CMC with inlet concentrations of 3–0.3–3 mM SDS, using *D*_DS–_ = 5.7 × 10^–10^ m^2^/s. Though this value of *D*_DS–_ is not the value which leads to the most positive diffusiophoretic mobility in the high-concentration range, it is illustrative for permitting a diversity of behaviors below the CMC, as can be seen in Figure 2C: a constant negative *M*_DP_ = −9.6 × 10^–11^ m^2^/s for ζ = −70 mV, a constant positive M_DP_ = 1.9 × 10^–10^ m^2^/s for ζ = −120 mV, and for ζ = ζ_fit_(*S*), a concentration-dependent *M*_DP_, which crosses over from negative to positive at *S*_X_ = 0.59 mM. From the predicted colloid concentration profiles, signatures of positive and negative diffusiophoretic mobilities in the channel geometry are established. The results for *D*_DS–_ = 5.7 × 10^–10^ m^2^/s with ζ = −70, −120 mV, and ζ_fit_(*S*) at *t* = 1 and 140 s are shown in Figure 4.

At early times before the solute concentration profile has relaxed significantly in the channel center, e.g., at *t* = 1 s, diffusiophoresis has a localized effect at each solute front, which reveals clear signatures of the direction of diffusiophoretic transport. For a constant negative diffusiophoretic mobility such as in the case where ζ = −70 mV (Figure 4A), two maxima in the colloid profile occur in the central region, flanked by two minima on the outside, due to inward-directed diffusiophoretic transport toward the central region of the lower solute concentration. For a constant positive diffusiophoretic mobility, as in the case where ζ = −120 mV (Figure 4B), the relative positions of the maxima and minima are switched: outward transport toward higher solute concentrations leads to two minima in the central region flanked by maxima outside. For a diffusiophoretic mobility that changes sign across the solute gradient, as in the case where ζ = ζ_fit_(*S*) (Figure 4C), the colloid profile displays two minima that are each surrounded by two local maxima due to the combined behavior of the positive and negative regions. At the location where the mobility changes sign, diffusiophoretic transport propels particles out of the minima in both directions, resulting in two local maxima, one on each side of the crossover point. This feature is a signature of a diffusiophoretic mobility that changes sign at an intermediate concentration in the concentration gradient.

At later times, the profiles can be understood as the accumulation of the initial trends, with the innermost local extrema from early times combining into a single local extremum in the channel center due to relaxation of the solute gradient into that region. Therefore, at later times, the strictly negative mobility yields a central maximum flanked by regions of colloid depletion on both sides (Figure 4D) and the strictly positive mobility yields the opposite behavior (Figure 4E). At later times, the characteristic of a reversing mobility from negative to positive is a profile consisting of a local maximum, which accumulates in the channel center due to the meeting of the inner maxima, flanked by two minima, which are in turn flanked by two more local maxima (Figure 4F). By identifying these signatures in the colloid profiles, the sign of the diffusiophoretic mobility, including whether or not it has reversed sign across the channel, can be determined clearly.

**Figure 5:**
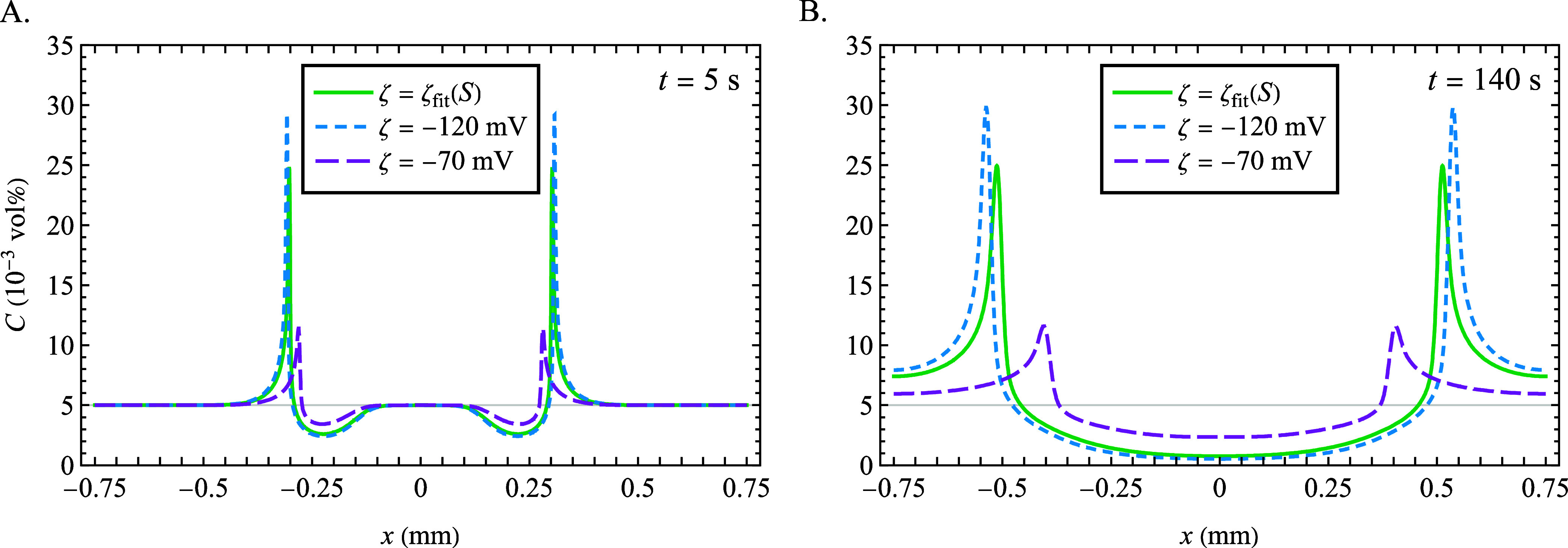
Numerically computed colloid concentration profiles at (A) *t* = 5 s and (B) *t* = 140 s for solute concentration gradient 300–30–300 mM SDS, entirely above the CMC with *M*_DP_ > 0, for ζ = ζ_fit_(*S*) (green), ζ = −120 mV (blue), and ζ = −70 mV (purple). Inputs of *S*_cmc_ = 8.2 mM, *n* = 60, θ = 0.84, *D*_DS-_ = 3.9 × 10^–10^ m^2^/s, and *D_m_* = 1.0 × 10^–10^ m^2^/s were used to generate the least positive *M*_DP_ in the SDS concentration range.

Finally, the diffusiophoretic transport above the CMC (inlet concentrations of 300–30–300 mM SDS) is simulated by solving eqs 10–13. The same three ζ-potential cases from above are used along with the set of parameters, which were found previously (*n* = 60, θ = 0.84, *D_m_* = 1.0 × 10^–10^ m^2^/s, and *D*_DS–_ = 3.9 × 10^–10^ m^2^/s) to enable the greatest propensity to generate a less positive mobility. The results are shown in Figure 5.

Since all justifiable parameters yield a positive mobility above the CMC, the expected behavior for the colloid profile with a positive diffusiophoretic mobility is produced in all cases: at *t* = 5 s (Figure 5A), two local minima form on the inner side of the solute front, flanked by maxima on their outer sides. At *t* = 140 s (Figure 5B), these minima combine into a central minimum, with two larger maxima on the outsides, whose sharpnesses are determined by the ζ-potential. Although these computations account for the concentration dependence in the mobility due to the changing fraction of micellization (and the changing ζ-potential when ζ = ζ_fit_(*S*) is used), the model does not predict a negative mobility anywhere in this concentration range, including that which would arise from a reversal from negative to positive sign of the diffusiophoretic mobility as was indicated below the CMC in Figure 4C,F. Next, experimental results of diffusiophoretic colloid transport in the microfluidic geometry will be reported, and numerically computed colloid profiles using analogous conditions will be compared.

### Experimental Results

**Figure 6:**
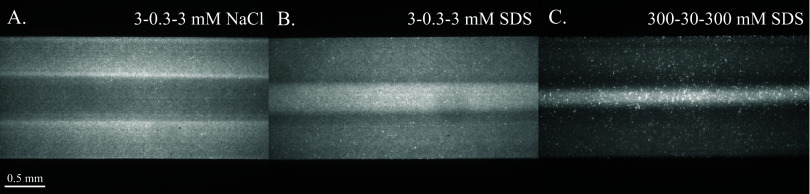
Averaged channel images of fluorescent colloids at *t* ∼ 120–150 s (left to right) for (A) 3–0.3–3 mM NaCl gradient; (B) 3–0.3–3 mM SDS gradient; and (C) 300–30–300 mM SDS gradient. *U* = 1.3 × 10^–4^ m/s and *t* = *z*/*U*.

Images in Figure 6 show the long-time behavior of colloids in various solute gradients. The left and right sides of each image correspond to *z* positions correlating to ∼120 and 150 s of transport, respectively (*U* = 1.3 × 10^–4^ m/s, where *t* = *z*/*U*). Each image is an averaged composite of 100 images recorded 1 s apart. In each case, the colloid was introduced in all three inlets at a concentration of 0.005 vol %, so its initial condition is a uniform concentration across the channel. All three of the solute gradient cases have the same initial magnitude of the gradient of ln *S*. Figure 6A shows the colloid behavior in response to a solute gradient of 3–0.3–3 mM NaCl (concentration values corresponding to the outer, inner, and outer inlets). It has been well established that colloidal particles with negative ζ-potentials exhibit positive diffusiophoretic mobility in gradients of NaCl and thus migrate toward regions of higher NaCl concentration.^18,21,32^ This is shown in Figure 6A where the fluorescence intensity, proportional to colloid concentration, was weakest in the center (appearing dark), where NaCl concentration was the lowest, and two prominent intensity bands (appearing bright) appeared outside of the central region indicating colloidal migration toward high-solute concentration regions. The prominent intensity bands, indicating local colloid accumulation, form close to the regions where the high- and low-solute regions meet because this is where the solute gradient is the largest. This NaCl result demonstrates the capacity of this experimental configuration to detect evidence of positive diffusiophoretic mobility. To confirm that the detection of the mobility is insensitive to the direction of the solute gradients established in the microfluidic device, an additional experiment was conducted with NaCl, reversing the gradient to place the high concentration in the center rather than the outer sides of the channel. The colloid transport direction was indeed reversed in the device as expected, as the colloids migrated inward to accumulate in the center where the NaCl concentration was highest, as opposed to migrating outward when the NaCl concentration was highest in the outer sides.

Figure 6B,C shows the colloid behavior in solute gradients of 3–0.3–3 mM SDS and 300–30–300 mM SDS, respectively. All SDS concentrations in the channel in Figure 6B are below the 8.2 mM CMC, and all concentrations in the channel in Figure 6C are well above the CMC and above the highest concentration *S*_X_ = 25.9 mM at which the model predicted a positive mobility for reasonable input parameters.

There is no region in the low SDS concentration experiment where micelles would be present, and no region in the high SDS concentration experiment where micelles would not be present. Both Figure 6B,C show the greatest colloid concentrations (appearing as bright regions) in the central region of the channel, bounded by narrow regions of low concentration (appearing dark). There is a depletion of colloids close to the regions where high and low SDS concentration regions meet where the solute gradient is the largest, meaning the colloids were most strongly repelled from high SDS regions there in both cases. Notably in Figure 6C, where the solute gradient was established above the CMC, there is more prominent narrowing and depletion of colloids than in Figure 6B where the solute gradient was below the CMC. For both cases, the narrowing and depletion of colloids from high-solute regions show the migration away from high-solute regions and are indicative of a negative diffusiophoretic mobility.

The bright spots scattered around Figure 6C are colloids that have adhered to the channel wall and become immobile at higher SDS concentrations. The number of adherent particles increased gradually as the actual duration of the experiment increased (actual time, as opposed to effective time *t* = *z*/*U*). These immobilized particles had no impact on the bulk colloidal transport behavior, however, as colloid concentration profiles with few adherent particles gathered shortly after starting an experiment were consistent with those gathered after long durations with more numerous adherent particles. The adherent particles simply contributed localized “sharp spikes” in the profiles.

**Figure 7:**
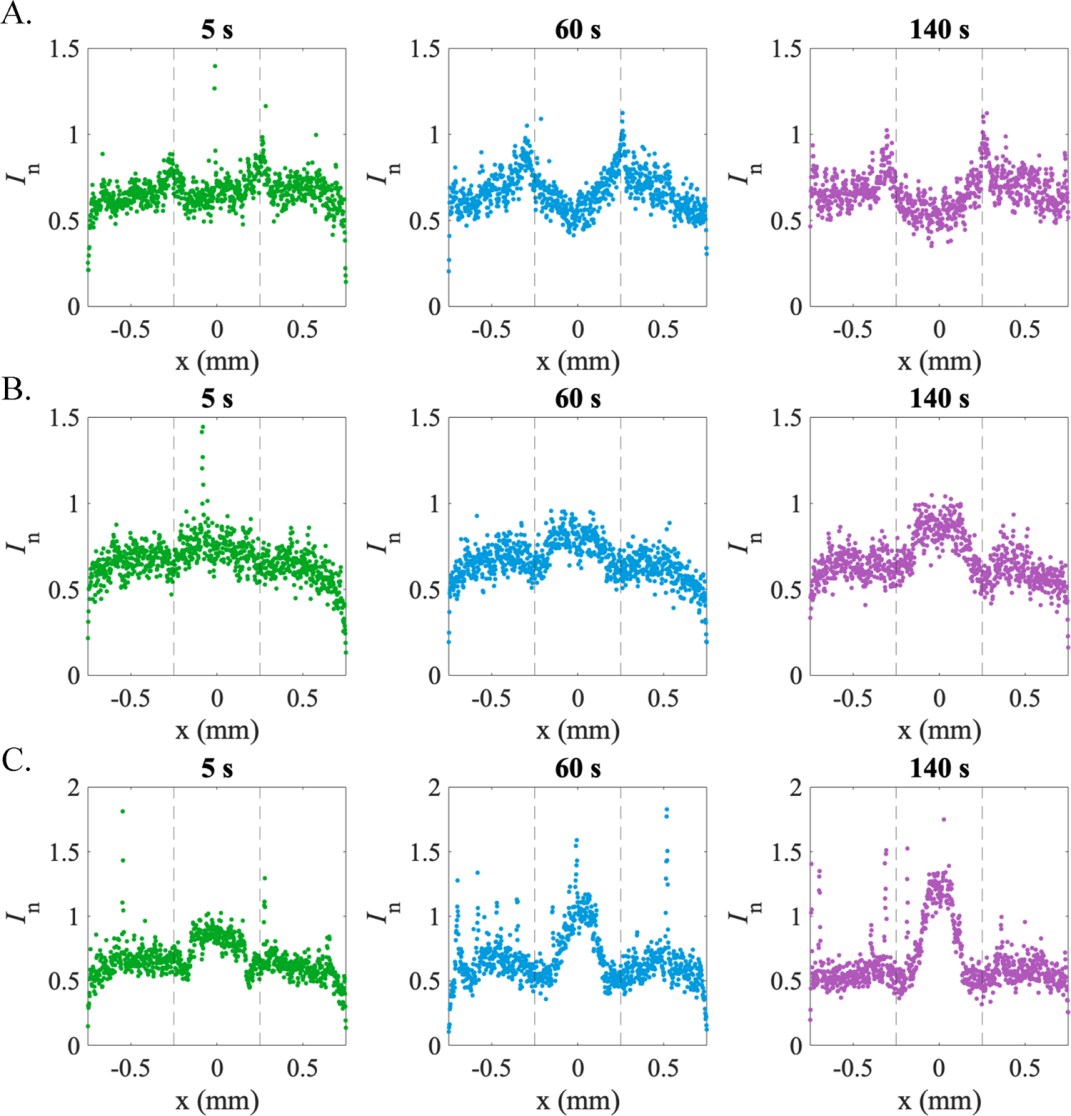
Normalized intensity, *I*_n_, line scans across the channel (*x*-direction) for *t* = 5, 60, and 140 s for (A) 3–0.3–3 mM NaCl gradient; (B) 3–0.3–3 mM SDS gradient; and (C) 300–30–300 mM SDS gradient. The dashed lines are the boundaries of the central inlet position where the high- and low-solute regions initially meet.

To facilitate the comparison of experimental results with the numerical model, Figure 7 shows line scans of normalized intensity, *I*_n_, for transport times of 5, 60, and 140 s for all three solute gradient cases. For the NaCl gradient (Figure 7A), the line scans showed the evolution of a single central minimum bounded by two maxima, signifying a positive diffusiophoretic mobility. Both SDS gradients (Figure 7B,C) produced a single central maximum surrounded by two narrow minima, becoming more pronounced as transport time increased, indicative of a negative diffusiophoretic mobility. This indicates that the diffusiophoretic mobility was negative at all concentrations examined below and above the CMC, with no sign of a positive mobility, contrary to predictions of the existing theory of diffusiophoresis in micellar SDS solutions. Furthermore, the evolution of the colloid distribution in the manner expected for negative diffusiophoretic mobility was more pronounced above the CMC than below. Since the micelle concentration varies far more significantly than the monomer concentration across the gradient when all concentrations exceed the CMC, this result suggests a significant capacity for micelles to drive diffusiophoresis.

**Figure 8:**
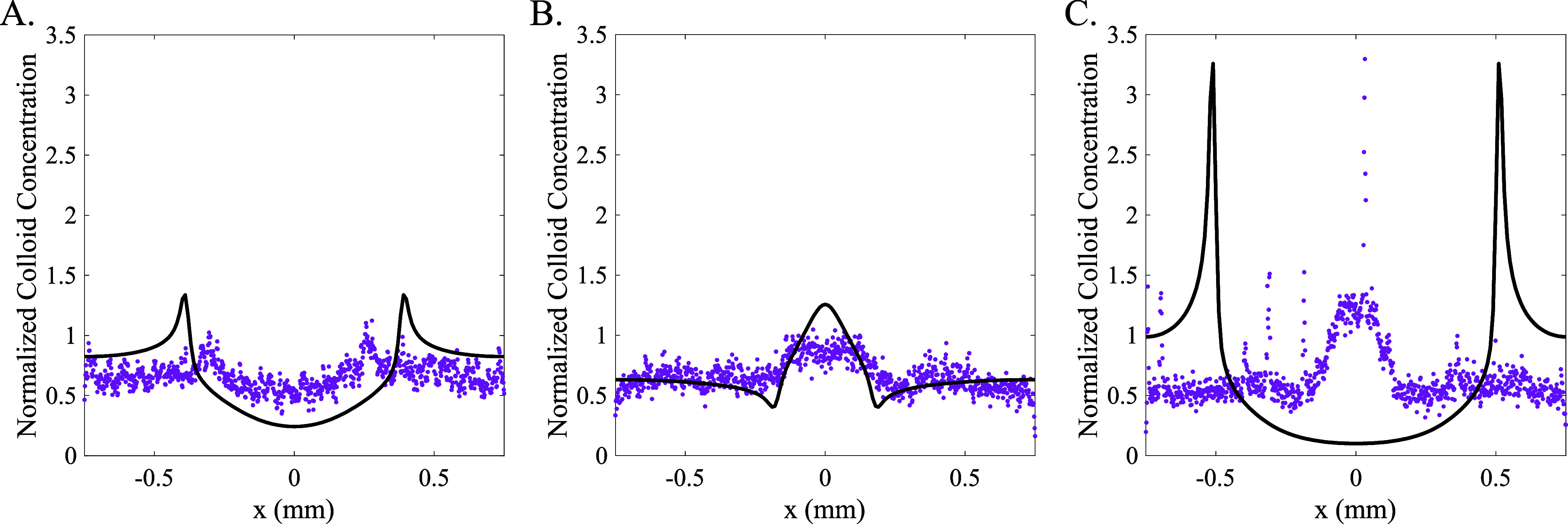
Experimental (dotted) and numerical (solid) normalized colloid concentration profiles at *t* = 140 s for (A) 3–0.3–3 mM NaCl gradient, numerical profile using ζ = ζ_fit_(S) for NaCl; (B) 3–0.3–3 mM SDS gradient using ζ = ζ_fit_(*S*) for SDS and *D*_DS_^–^ = 3.9 × 10^–10^ m^2^/s; (C) 300–30–300 mM SDS gradient using ζ = ζ_fit_(*S*) for SDS, *S*_CMC_ = 8.2 mM, *n* = 60, θ = 0.84, *D*_DS_^–^ = 3.9 × 10^–10^ m^2^/s, and *D_m_* = 1.0 × 10^–10^ m^2^/s.

Figure 8 shows the normalized intensity of the colloids in the channel compared with the numerically computed normalized colloid concentration profile at 140 s for all three solute cases. The numerically computed colloid concentration profiles were normalized by the total area under the colloid concentration curve at each time to facilitate comparison with the experimental data. Calculations for the NaCl gradient (Figure 8A) utilized *D*_Na_^+^ = 1.3 × 10^–10^ m^2^/s and *D*_Cl_^–^ = 2.0 × 10^–9^ m^2^/s along with the fitted ζ-potential function (eq 14) for NaCl. There are quantitative discrepancies between the numerical and experimental colloid distributions, but the relative shapes for the experimental and predicted colloid profiles (a global minimum flanked by two maxima) are mutually consistent and as expected for a positive diffusiophoretic mobility.

For the SDS gradient entirely below the CMC (Figure 8B), the model using *D*_DS_^–^ = 3.9 × 10^–10^ m^2^/s and the fitted ζ-potential function (eq 14) for SDS predicted the diffusiophoretic mobility to be strictly negative within the 3–0.3–3 mM SDS gradient. In the numerical profile, this is reflected by a central maximum surrounded by two minima. This behavior is present in the experimental results. While the experimental signal resolution does not allow inspection for subtle variations that might indicate a variation of diffusiophoretic mobility with local SDS concentration below the CMC, the overall behavior is dominated by a negative diffusiophoretic mobility. Therefore, the predicted diffusiophoretic mobility using *D*_DS_^–^ = 3.9 × 10^–10^ m^2^/s and ζ = ζ_fit_ (*S*) for SDS agrees in sign with the experimental results below the CMC.

Above the CMC (Figure 8C), the experimental results are in sharp qualitative disagreement with the model-predicted numerical colloid profile using ζ = ζ_fit_ (*S*) for SDS. The set of parameters n = 60, θ = 0.84, *D*_DS–_ = 3.9 × 10^–10^ m^2^/s, and *D_m_* = 1.0 × 10^–10^ m^2^/s were chosen to give the least positive values of *M*_DP_ in the range of 30–300–30 mM SDS. Despite this, the model still predicted a strictly positive mobility in the experimental concentration range (300–30–300 mM SDS), and no other parameters, even a constant ζ-potential of ζ = −70 mV (lower than any value measured in the current system), would permit *M*_DP_ to be negative anywhere in this SDS concentration range (see Figure 2).

This strictly positive mobility predicted by the numerical simulation is manifested in the appearance of a global minimum at the center bounded by two sharp outer maxima in Figure 8C. In contrast, the experimental results show a single sharp central maximum bounded by two minima. This is the definitive signature of a negative diffusiophoretic mobility where the colloids are migrating toward the channel center, away from the high-solute regions. This shows that the colloid evolution was evidently dominated by a negative diffusiophoretic mobility across the SDS concentration range from 30 to 300 mM. To consider the possibility of a positive mobility for a small range of sufficiently high SDS concentrations (meaning the sign of *M*_DP_ changes within the gradient), the experimental profile would have a set of small local maxima on the outer edges of the minima (see Figure 4F). However, no significant maxima are discernible within the resolution of the experimental signal. Therefore, the experimental results do not give any indication of a positive mobility in the concentration range of 30–300 mM SDS.

#### Flow Effects in the Channel

While the transport model assumes a uniformity of flows and colloid concentrations throughout the channel depth in the experiment, two effects could potentially challenge this assumption. First, as in similarly designed experiments,^18,33,34^ the parabolic flow profile of flow in the *z*-direction as it varies across the channel depth (*y*-direction) has the form *U*_p_ (*y*) = *Uy*(*d* – *y*)/6*d*^2^, where *d* = 85 μm is the channel depth. In the absence of diffusion, this leads to a variation in residence times across the channel depth, where the axial velocity has a variance of 44% of the mean velocity *U*. Diffusion across the depth will soften these variations. For the solute, the corresponding Peclet number *Ud*/*D*_s_ ≈ 4–7 is relatively small and diffusion in the *y*-direction is significant, leading to more uniformity in the solute concentration across the depth of the channel. However, due to the smaller diffusion coefficient of the colloidal particles, their corresponding Peclet number *Ud*/*D*_p_ ≈ 10^4^ and the colloids do not diffuse very far in the *y*-direction before leaving the microfluidic channel. As a result, experimental profiles of colloid concentrations are effectively averaged across different times. This effect could explain quantitative discrepancies between the experiments and the transport model but would not affect the qualitative observation of consistent negative mobility diffusiophoretic transport in gradients of SDS.

A second flow effect, which could be present in the channel, is diffusio-osmotic flow driven at the channel walls. Recent studies^33,34^ have shown how this effect can lead to convective cells in the channel cross section and advection of colloidal particles near the channel walls. The theoretical expression for the diffusio-osmotic mobility *M*_DO_, giving the fluid slip velocity at the channel wall via *u*_slip_ = −*M*_DO_∇ ln *S*, is theoretically equivalent to *M*_DP_ because of the thin-layer limit. In this case, diffusio-osmotic advection would drive the transport of particles near the walls in the opposite direction of their diffusiophoresis, leading to an apparent superimposition of particle broadening and narrowing in channel images, as observed by Chakra and co-workers and Migacz and co-workers.^33,34^ However, in the current experiments, there was no evidence of simultaneous inward (narrowing) and outward (broadening) colloid transport—as could have been driven by either diffusio-osmotic advection or by a concentration-dependent crossover in the sign of *M*_DP_. Therefore, a significant influence of diffusio-osmotic advection can be ruled out in the current study, perhaps due to the larger channel depth in the current microfluidic device compared to the devices used in those other studies. One may also consider if, on the other hand, a difference in ζ-potentials of the particles and the channel walls led to a relative crossover in mobility between the signs of *M*_DO_ and *M*_DP_. If this occurred, then diffusio-osmosis would be collaborating in driving colloid transport in the same direction as diffusiophoresis. In either case, the qualitative observation that gradients of SDS drive transport consistent with a negative *M*_DP_ above and below the CMC is unaffected.

#### Implications for the Role of Micelles in Diffusiophoresis

The observation that the diffusiophoretic mobility of the negatively charged polystyrene colloids in SDS gradients was consistently negative for concentration gradients that were either entirely below or entirely above the CMC is in contradiction to the current conceptual understanding of the role of micellization in diffusiophoresis, in which micelles are proposed to play an insignificant role. The results suggest that gradients of micelles and their counterions contribute to the diffusiophoretic mobility as strongly as, if not more strongly than, monomeric surfactants in the same initial ln *S* gradient. When all concentrations are above the CMC, the variation in monomeric surfactant concentration is weak, and the gradient is dominated by the varying micelle concentration. The occurrence of diffusiophoretic transport that was comparable to, in fact greater than, the extent that occurred in a nonmicellar gradient suggests a potent role for micelles and their counterions.

The current theoretical model^13^ was the first to address micellization effects in diffusiophoresis. The current experimental results indicate that this model predicts an overly positive mobility at high SDS concentrations, as the negative electrophoretic contribution *M*_EP_ remains dominant over the positive chemiphoretic contribution *M*_CP_. Here, we consider possible sources of discrepancy that may suggest model refinements.

One possibility is that ζ-potentials calculated from experimentally measured electrophoretic mobilities neglect factors such as polarization effects, as noted above. This is unlikely to be an important source of discrepancy since the treatment of the electrophoretic component of the diffusiophoretic mobility makes precisely the same model assumptions as were made here (Smoluchowski equation and neglect of polarization effects). As a result, the ζ-potential measurements used here as model inputs directly indicate the electrophoretic response of the colloidal particle to a known electric field. Using these measured values of the ζ-potential to calculate *M*_EP_ thus has a high level of confidence, with uncertainty confined to the model of the diffusion-generated bulk electric field. Therefore, it seems the discrepancy between the model and the experiments may be traceable to an overly high prediction of *M*_CP_. Certain assumptions made in the model, such as the complete exclusion of micelles from the double layer, micelle monodispersity, fast micellization kinetics whereby micelles, counterions, and surfactant monomers are always in dynamic equilibrium, and neglect of nonelectrostatic interactions such as excluded-volume interactions, could be revisited. Furthermore, if one were to allow micelles to be present in the double layer, then one could introduce the dependence of micelle aggregation number and counterion binding on the local electrostatic potential.

## Conclusions

Prior experimental investigation of diffusiophoresis of charged colloids in ionic surfactant gradients had not completely isolated the effect of micellization on diffusiophoresis, and it had been proposed that micelles played an unimportant role. A theoretical model motivated by these observations predicted a transition from negative to positive diffusiophoretic mobilities for negatively charged colloids with increasing concentration of anionic surfactants. Here, surfactant solute gradients and a favorable configuration of solute and colloid concentrations in a microfluidic channel were designed to investigate diffusiophoresis both in the complete absence of micelles and in the presence of micelles and to detect either positive or negative diffusiophoretic mobility. Microfluidic transport experiments of negatively charged colloids undergoing diffusiophoresis in anionic surfactant gradients showed significant diffusiophoretic transport consistent with negative diffusiophoretic mobility values both wholly above and wholly below the critical micelle concentration of the anionic surfactant, sodium dodecyl sulfate.

Numerical modeling was used to predict the colloid profiles in such experiments. The modeling used existing theory to incorporate the effects of micellization, and it used experimental ζ-potential measurements over a range of solute concentrations to account for the variable diffusiophoretic mobility that arises with a ζ-potential that is a function of solute concentration.^13,15^ A sensitivity analysis of the numerical results was performed to better understand the impacts of uncertainty in measured input parameters, with the conclusion that the existing theory consistently predicted the appearance of positive diffusiophoretic mobility in the current system above the CMC, for any reasonable values of input parameters.

While the current experiments agree with previous studies conducted below the CMC in terms of the direction and relative magnitude of transport,^7−10^ the current experiments conducted entirely above the CMC here show significant colloid transport with signatures of only a negative diffusiophoretic mobility and no reversal in sign anywhere in the gradient. This contradicts the theoretical prediction of a positive diffusiophoretic mobility above the CMC.^13^ The strong diffusiophoretic transport measured above the CMC here also contrasts with previous experimental work^7^ where only weak transport was detected in microfluidic devices using semipermeable membranes to establish the surfactant concentration gradient. The primary conclusion of the current work is that ionic surfactant micelles and their counterions contribute significantly to the diffusiophoresis of similarly charged colloids in a manner that is not yet captured by existing theory.

## Supporting Information Available

Table of predicted mobility crossover concentrations calculated using different input parameters (PDF)
