# Supplementary material for: Diffusiophoretic Transport of Charged Colloids in Ionic Surfactant Gradients Entirely below versus Entirely above the Critical Micelle Concentration
Source: Langmuir. 2024 May 1;40(19):10143–56. doi: 10.1021/acs.langmuir.4c00431 (PMC11100018; doi:10.1021/acs.langmuir.4c00431)
Supplement: Supplementary file 1 — la4c00431_si_001.pdf [file la4c00431_si_001.pdf]

Supporting Information

Diffusiophoretic transport of charged colloids in ionic surfactant gradients entirely below versus entirely above the critical micelle concentration

Angela Yang[1#], Brian E. McKenzie[1#], Benjamin Pavlat[1], Eric S. Johnson[2], Aditya S. Khair[1], Stephen Garoff[3], Robert D. Tilton[1, 4*]

[1]Department of Chemical Engineering, Carnegie Mellon University, Pittsburgh, PA 15213

[2]The Procter & Gamble Company, Cincinnati, OH, USA 45241

[3]Department of Physics, Carnegie Mellon University, Pittsburgh, PA 15213

[4]Department of Biomedical Engineering, Carnegie Mellon University, Pittsburgh, PA 15213

*****Corresponding author: Department of Chemical Engineering, Carnegie Mellon University, 5000 Forbes Avenue, Pittsburgh, PA 15213, USA. Tel: 1-412-268-1159. Email: tilton@cmu.edu

[#] Angela Yang and Brian E. McKenzie contributed equally to this work.

Table S1: Diffusiophoretic mobility crossover concentrations under tested conditions

S_{CMC} (mM)	n	θ	$D_{\mathrm{Na}+}$ $\left(10^{-10}\frac{\mathrm{m}^2}{\mathrm{s}}\right)$	$D_{\mathrm{DS}-}$ $\left(10^{-10}\frac{\mathrm{m}^2}{\mathrm{s}}\right)$	D_{m} $\left(10^{-10}\frac{\mathrm{m}^2}{\mathrm{s}}\right)$	ζ (mV)	S_{X} (mM)
Variation with micelle properties (Figure 2A)							
8.2	60	0.8	13	3.9	1.0	$\zeta_{\mathrm{fit}}(S)$	15.7
8.2	60	0.7	13	3.9	1.0	$\zeta_{\mathrm{fit}}(S)$	10.2
8.2	60	0.6	13	3.9	1.0	$\zeta_{\mathrm{fit}}(S)$	9.1
8.2	80	0.8	13	3.9	1.0	$\zeta_{\mathrm{fit}}(S)$	12.5
8.2	80	0.7	13	3.9	1.0	$\zeta_{\mathrm{fit}}(S)$	9.3
8.2	80	0.6	13	3.9	1.0	$\zeta_{\mathrm{fit}}(S)$	8.8
8.2	100	0.8	13	3.9	1.0	$\zeta_{\mathrm{fit}}(S)$	10.9
8.2	100	0.7	13	3.9	1.0	$\zeta_{\mathrm{fit}}(S)$	9.0
8.2	100	0.6	13	3.9	1.0	$\zeta_{\mathrm{fit}}(S)$	8.6
Variation with solute self-diffusion coefficients (Figure 2B)							
8.2	60	0.8	13	5.7	1.0	$\zeta_{\mathrm{fit}}(S)$	14.8
8.2	60	0.8	13	5.7	1.1	$\zeta_{\mathrm{fit}}(S)$	13.5
8.2	60	0.8	13	5.7	2.1	$\zeta_{\mathrm{fit}}(S)$	0.59
8.2	60	0.8	13	5.2	1.0	$\zeta_{\mathrm{fit}}(S)$	15.0
8.2	60	0.8	13	5.2	1.1	$\zeta_{\mathrm{fit}}(S)$	13.8
8.2	60	0.8	13	5.2	2.1	$\zeta_{\mathrm{fit}}(S)$	1.2
8.2	60	0.8	13	3.9	1.0	$\zeta_{\mathrm{fit}}(S)$	15.7
8.2	60	0.8	13	3.9	1.1	$\zeta_{\mathrm{fit}}(S)$	14.5
8.2	60	0.8	13	3.9	2.1	$\zeta_{\mathrm{fit}}(S)$	9.4
Variation with zeta potential and dodecylsulfate self-diffusion coefficient (Figure 2C)							

8.2	60	0.8	13	5.7	1.0	$\zeta_{\text{fit}}(S)$	14.8
8.2	60	0.8	13	5.7	1.0	-120	12.4
8.2	60	0.8	13	5.7	1.0	-70	25.9
8.2	60	0.8	13	5.2	1.0	$\zeta_{\text{fit}}(S)$	15.0
8.2	60	0.8	13	5.2	1.0	-120	13.0
8.2	60	0.8	13	5.2	1.0	-70	25.9
8.2	60	0.8	13	3.9	1.0	$\zeta_{\text{fit}}(S)$	15.7
8.2	60	0.8	13	3.9	1.0	-120	14.0
8.2	60	0.8	13	3.9	1.0	-70	25.7
